# Internal consistency and structural validity of the parent-report preschool (2-4 years) Strengths and Difficulties Questionnaire in 1-year-old children

**DOI:** 10.1186/s41687-025-00905-1

**Published:** 2025-06-23

**Authors:** Sarah L. Blower, Kate E. Mooney, Nicole Gridley, Fionnuala Larkin, Tracey J. Bywater, G. J. Melendez-Torres

**Affiliations:** 1 Department of Health Sciences, Seebohm Rowntree Building, University of York, York, YO10 5DD UK; 2 Carnegie School of Education, Carnegie Hall, 222, Headingley Campus, Leeds Beckett University, Leeds, LS1 3HE UK; 3 School of Applied Psychology, University College Cork, Cork Enterprise Centre, North Mall, Cork, Ireland; 4 University of Exeter Medical School, South Cloisters, University of Exeter, St Luke’s Campus, Heavitree Road, Exeter, EX1 2LU UK

## Abstract

**Background:**

Prevention and early intervention are key to addressing poor child mental health. Systematic reviews have highlighted a lack of brief, valid and reliable outcome measures that can be implemented in both research and practice to assess social, emotional and behavioural outcomes in the early years. The Preschool Strengths and Difficulties Questionnaire (2–4 years) is a promising candidate to fill this gap, but the measurement properties of this tool are not yet known in very young children.

**Methods:**

A secondary data analysis of two clinical trial datasets was conducted to examine the internal consistency reliability and structural validity of the parent-report English preschool version of the Strengths and Difficulties Questionnaire in a sample of 505 infants with mean average age of 18 months (SD .81). The measure was designed for children aged 2–4 years and was not modified prior to use with 1-year-olds in this study. Structural validity was examined in two Confirmatory Factor Analyses (CFA) testing two-factor and five-factor models (representing factor structures proposed by the developers of SDQ), and McDonald’s coefficient Omega was estimated for each subscale with values > .70 considered acceptable.

**Results:**

The model fit values for the two-factor model demonstrated a poor fit to the data (*X*^*2*^ = 626.067(151) = *p* < .001, CFI = 0.612, RMSEA = 0.079 [90% CI .073 to .085], SRMR = .077) and the omega value was below acceptable at ω = .57 for the internalising subscale and ω = .76 for the externalising subscale. The five-factor model also demonstrated a poor fit to the data (*X*^*2*^ = 836.813(242) = *p* < .001, CFI = 0.676, RMSEA = 0.070 [90% CI .065 to .075], SRMR = .081). Omega values were below acceptable for three out of five subscales.

**Discussion:**

We concluded that the measure has poor internal consistency and lacks structural validity in this very young age group. Further research to adapt the SDQ in order to improve content and face validity is recommended prior to any further psychometric analyses with this very young age group. The paucity of robust and practical outcome measures of early social, emotional and behavioural poses significant challenges to the early identification of need and evaluation of interventions.

**Supplementary Information:**

The online version contains supplementary material available at 10.1186/s41687-025-00905-1.

## Background

Prevention and early intervention to address emotional and behavioural difficulties in early childhood are key to reducing negative emotional, behavioural, social difficulties later in life [1]. Dimensional measures of emerging symptomatology throughout childhood, including infancy, are needed to understand developmental change and support the early identification of needs and evaluate interventions [2, 3]. Systematic reviews [4–6] have identified an increasing choice of parent-report measures for assessing emotional and behavioural outcomes in infancy, though many have access restrictions and licensing costs, and limited evidence of longitudinal and short-term utility, validity and reliability [7]. Reviewers highlight the need to further evaluate existing measures before developing new ones.

SDQ is a 25-item instrument intended as a screen for emotional and behavioural difficulties in children [8]. The SDQ is well known in child and family outcome research and practice [3, 9]. It has the capacity to measure outcomes across multiple ages and development stages, is translated into multiple languages, is available in both parent and teacher report, does not require a clinical license, and is free to download and use. For these reasons the ‘Pre-school’ version of SDQ (for children aged 2–4 years) was selected as a secondary outcome measure in the ‘Enhancing Social and Emotional Health in the Early Years’ randomised controlled trial (E-SEE study) which provides the premise for this report [10].

Robust evidence of the psychometric properties of School Age SDQ (for children aged 4–14) has been reported [4, 11]. However, the preschool SDQ is a modified downward extension of the School Age SDQ, few studies have explored the measurement properties of this tool and it was not developed specifically for very young children. In a study of 16,659 children aged 3–4 years, Croft et al. [12] reported acceptable internal reliability scores for all scales (ω range = 0.75–0.82 for 3-year-olds) with the exception of peer problems (ω = 0.66) and following a confirmatory factor analysis (CFA) reported acceptable model fit for a five factor structure originally proposed by Goodman [13] (*X*^*2*^ = 28,332(2520) = *p* < .0005, CFI = 0.905, RMSEA = 0.025). D’Souza et al. reported acceptable model fit following CFA for a modified five-factor structure (*X*^*2*^ = 3361.02(260), CFI = 0.905, RMSEA = 0.047, TLI = 0.891) and internal consistency for all subscales (α range = 0.71–0.84) except peer problems (α = 0.54) in a sample of 2-year-olds [14]. A study of the German language version of preschool SDQ with 1,738 children aged 3–5 years [15] also reported a CFA of the original five-factor structure proposed by Goodman, with RMSEA (0.049) and GFI (0.934) indicating a good fit, but CFI (0.859) not reaching acceptable thresholds.

Only one study to date has evaluated the measurement properties of preschool SDQ in 1–2 year olds. Patel et al. reported good levels of concurrent validity between subscales of the preschool SDQ with corresponding subscales on the Child Behaviour Checklist (r range = 0.19 to 0.57) and moderate levels of internal consistency reliability for the “Hyperactivity,” (α = 0.67) “Prosocial,” (α = 0.79) and “Externalizing” subscales (α = 0.65) only [3]. The study suggests promising evidence for the SDQ in this age group but is limited by a small sample size (n = 93). Further research with larger, more diverse, samples is needed to explore how well the factor structure of the measure operates in very young children.

Despite the need for robust measures of behavioural and emotional development in infants under 2 years of age, there is a paucity of studies evaluating the measurement properties of brief, accessible tools such as preschool SDQ. The current study uses secondary data from E-SEE to examine the internal consistency and structural validity of the English preschool SDQ in a sample of 1-year-olds. Due to the extensively characterised five factor structure of the SDQ in its target populations and our objective to understand scale performance over a younger age range, we applied CFA with the five-factor model representing the five strengths and difficulties identified by SDQ developers [8, 13]. We also conducted CFA with a two-factor model that is less well established in the literature and that represents broader internalizing and externalizing SDQ subscales considered to have more practical utility in low-risk samples, whereas the five subscales are more useful when screening for disorder [16].

## Methods

### Participants and procedures

The E-SEE Study (ISRCTN11079129) was a randomised controlled trial of a proportionate universal parenting intervention. The main trial results showed the intervention was not effective; there were no significant differences between arms on adjusted mean difference scores for all primary and secondary and outcomes.

Data from E-SEE’s external pilot and main trial phase, from both the intervention and control arm participants, are included in the current study. Different individuals were recruited in the pilot and main trials. E-SEE study design is detailed in the study protocol [17], pilot results [18] and main results papers [10].

Mothers of typically developing infants aged 8 weeks or younger were recruited from five sites in England, and self-referred or referred by health or family practitioners. Mothers gave informed, written consent to participate. Participant data was collected in the home context by trained researchers. The last date of E-SEE data collection was February 2020 i.e. prior to the Covid-19 pandemic and lock-downs. A bespoke social and demographic form captured age, ethnicity, religion, income, marital status and parent/co-parent education.

### The preschool SDQ

All 25 items are rated on a 3-point Likert scale (0 = not true; 1 = somewhat true; 2 = certainly true) and respondents are asked to give their answers on the basis of the child’s behaviour over the last six months. The questionnaire includes 10 positively worded items (all items for the prosocial behaviour subscales and five reverse-scored items from the difficulties subscales), with all other items worded negatively. The SDQ comprises four difficulties subscales (emotional symptoms, peer problems, hyperactivity–inattention and conduct problems), and a strengths-focused prosocial behaviour subscale. Examples of items include: ‘helpful if someone is hurt, upset or feeling ill’ and ‘often unhappy, down-hearted or tearful’. A measure of total difficulties is calculated by summing all difficulties subscale scores. Higher scores indicate greater severity of difficulties for the difficulties subscales and total difficulties score, and greater strengths for the prosocial scale. The conduct and hyperactivity scale scores can be summed to obtain an ‘Externalising’ score, and the emotional and peer problems scales can be summed to produce an ‘Internalising’ score. More information about the measure can be found on the SDQ website (https://www.sdqinfo.org/).

### Statistical analyses

Data cleaning was conducted in Stata-18 [19] and statistical analyses was conducted in RStudio [20] using the *lavaan* package [21]. Children were included if they had a SDQ total difficulties score available, which was calculated if at least 3 out of the 4 subscales had scores. For further information on how missing data was handled, see the E-SEE SAP [22].

To assess internal consistency we estimated McDonald’s hierarchical Omega for each subscale, which conceptually reflects percentage of variance in the scale scores accounted for by a general factor. To assess structural validity two Confirmatory Factor Analyses (CFA) were run using a maximum likelihood estimator: (1) a two-factor model with ‘externalising’ (composed of the conduct and hyperactivity subscales) and ‘internalising’ (composed of the peer problems and emotional symptoms) subscales with the prosocial subscale dropped (in a simple one-order model without hierarchy), and (2) a five-factor model with the four difficulties subscales, and the prosocial behaviour subscale. For model fit, we report the Comparative Fit Index (CFI) (values > 0.90 are acceptable), Root Mean Square Error of Approximation (RMSEA) (values < 0.07 are acceptable), and Standardised Root Mean Square Residual (SRMR) (values < 0.08 are acceptable) [23]. Modification indices were inspected to investigate what amendments to the SDQ may improve the model fit. Factor loadings were considered to be statistically significant at p < 0.05.

## Results

### Participant descriptives and missing data

There were 644 participants total, after combining the main trial (n = 409) and pilot trial (n = 235) datasets. After excluding 90 co-parents and 49 participants without SDQ total difficulties scores (46 due to loss to follow-up, and 3 due to item level missingness meaning > 1 subscale could not be calculated), 505 participants remained. Of those who did complete the measure, 17 participants wrote comments on the margins of the paper form about the age appropriateness of the questionnaire, e.g. ‘too young for most of these questions’. Participant characteristics from the baseline questionnaire are reported in Table 1.

**Table 1:** Sample descriptives (n = 505)

Variable	
	**Mean (SD)**
**Parent age in years**	32 (4.9)
**Child age in months**	18 (.81)
	**Freq. (%)**
**Intervention group**	
Intervention	408 (81)
Control	97 (19)
**Parent ethnicity**	
White English/Welsh/Scottish/Northern Irish/British	375 (75)
Indian	35 (7)
Pakistani	35 (7)
Any other White background	26 (5)
All other ethnic groups combined	28 (5)
*Missing*	*6 (1)*
**Parent education**	
Higher education (PhD, Masters, Bachelors degrees, diplomas)	281 (56)
A-Levels	39 (7)
Compulsory/overseas/vocational qualifications	154 (30)
None of these qualifications	26 (5)
*Missing*	*5 (<1)*
**Parent relationship status**	
In a relationship	481 (95)
Not in a relationship	23 (5)
*Missing*	*1 (<1)*
**Child gender**	
Male	242 (48)
Female	257 (51)
*Missing*	*6 (1)*
**Child age in months at time of SDQ**	
16	2 (<1)
17	30 (6)
18	143 (28)
19	248 (49)
20	73 (14)
21	3 (<1)
*Missing*	*6 (1)*

Total difficulty scores ranged between 0–25, and the mean score was 9.32 (SD = 4.27). Scores did not significantly differ between the treatment and control group [10].

### Two-factor model

**Fig. 1:**
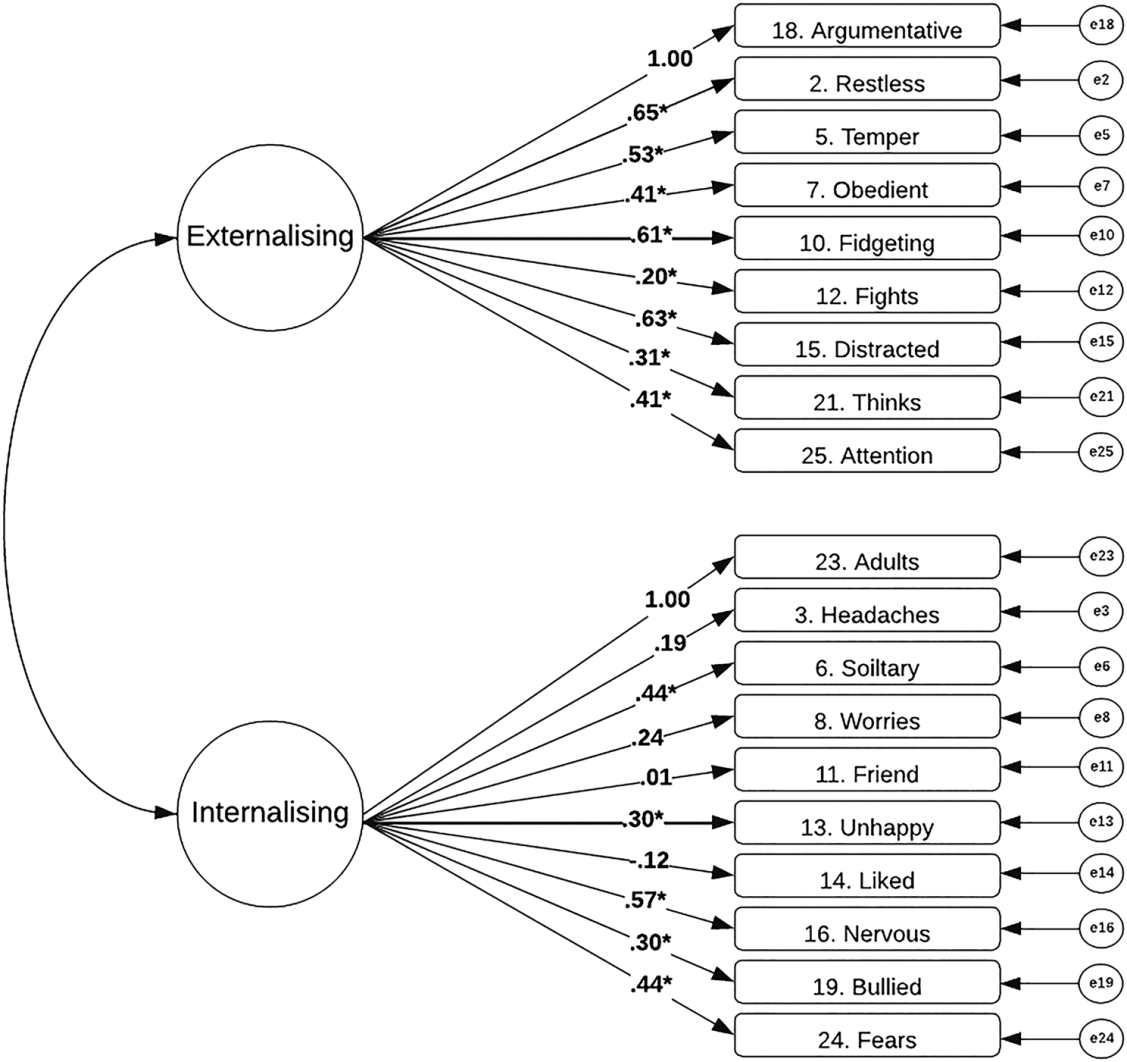
Model structure and standardised factor loadings with statistical significance (* = p < .05) for two-factor model

Figure 1 presents the model structure and factor loadings for the two-factor model of SDQ, with externalising and internalising subscales presented. The omega value was below acceptable at ω = 0.57 for the internalising subscale and ω = 0.76 for the externalising subscale. Factor loadings were all statistically significant for the externalising subscale, whereas factor loadings were only significant for five out of ten items in the internalising subscale. The model fit values for the two-factor model demonstrated a poor fit to the data (*X*^*2*^ = 626.067(151) = *p* < 0.001, CFI = 0.612, RMSEA = 0.079 [90% CI 0.073 to 0.085], SRMR = 0.077). Modification indices indicate that including residual correlations between some individual items would improve the model fit.

### Five factor model

**Fig. 2:**
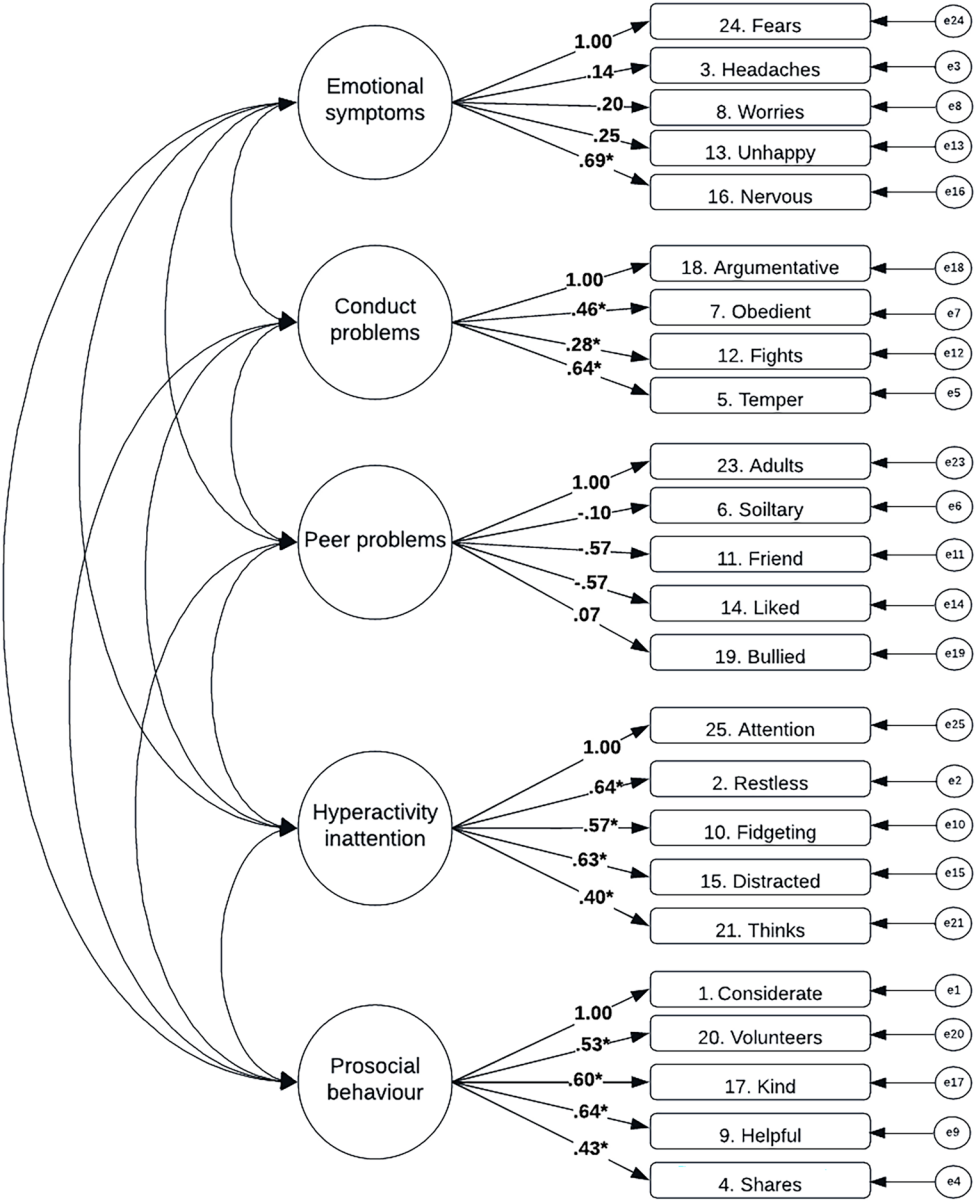
Model structure and standardised factor loadings loadings with statistical significance (* = p < .05) for five-factor model

Figure 2 presents the model structure and factor loadings for the five-factor model of SDQ, with the five subscales represented. Omega values were below acceptable for all subscales (ω = 0.71 for prosocial, 0.75 for hyperactivity, 0.37 for peer problems, 0.56 for conduct problems, 0.62 for emotional symptoms). The factor loadings for the prosocial, hyperactive, and conduct problems subscales were all significant, whereas factor loadings for emotional symptoms and peer problems were predominantly not significant. The model fit values for the five-factor model demonstrated a poor fit to the data (*X*^*2*^ = 836.813(242) = *p* < 0.001, CFI = 0.676, RMSEA = 0.070 [90% CI 0.065 to 0.075], SRMR = 0.081). Modification indices indicated that cross-loading items 25, 7, and 21 onto both the ‘prosocial’ and ‘peer problems’ subscales would improve the fit.

## Discussion

This is one of the first studies to explore the measurement properties of the pre-school SDQ in a sample of 1-year-old infants. Our analyses revealed that the measure has poor internal consistency and lacks structural validity in this age group. This is consistent with findings reported for many other widely favoured and implemented outcome measures for this young age range [6] though it is important to note that the pre-school SDQ was not originally designed to be used with children under 2 years.

There is some evidence that the conduct and hyperactivity (externalising) subscales are a better fit than subscales relating to internalising constructs (such as emotional difficulties) and peer relationships. This is consistent with previous research [3, 15] that reported moderate levels of internal consistency reliability for the key externalizing subscales on preschool SDQ in contrast to the internalizing subscales. Gustafsson et al. suggested that some emotional difficulties items may be more difficult to report on in children who have not yet developed verbal skills (e.g., headaches, stomach aches) [15]. It is also possible that externalising difficulties are easier to identify by parents and that in some cases internalizing items on the preschool SDQ may be developmentally inappropriate [3].

The preschool SDQ was designed to be used with children aged 2–4 years and our results reaffirm this guidance from SDQ developers (see SDQinfo – https://www.sdqinfo.org/). SDQ preschool was selected for inclusion in E-SEE because the children were approaching 2 years of age, due to the lack of other robust measures for this age range, and parental preference in public involvement activities undertaken during study design. However, the presence of item-level missing data on the SDQ compared to near 100% completion rates on all other measures in the study [10] taken together with annotations made by parents when completing the questionnaire suggests that the content validity of the preschool SDQ in 1-year-olds is likely to be weak.

On the basis of these findings, we cannot recommend the use of preschool SDQ with 1-year-olds in practice-based contexts (where it may be used to make decisions regarding the need for individualised support), nor in research contexts (where it may be used to assess the effectiveness of an intervention). Measures specifically designed to assess young children’s socio- emotional development may be more appropriate than SDQ, for example the Ages & Stages Questionnaire: Social-Emotional, Second Edition [24], though many of these measures have access restrictions and licensing costs that could limit their uptake in practice, particularly in low-resource contexts where versions in multiple languages are required.

In line with COnsensus-based Standards for the selection of health Measurement INstruments (COSMIN) guidelines [25], qualitative research involving cognitive interviewing techniques is recommended to explore and improve content validity of preschool SDQ in very young children. It is important to note that parents participating in E-SEE were almost all married or in long term co-habiting relationships (95%) and the majority were highly educated (56%). Further research exploring measurement invariance across different groups of caregivers in larger, more diverse samples is recommended. The use of maximum likelihood (ML) estimation assumes continuous, normally distributed indicators. Given that SDQ items are 3-point ordinal variables, this assumption may not fully hold. Although it is common practice to treat Likert-type scales as continuous in large-sample CFA, future work may benefit from using estimators designed for ordinal data. We also call for greater investment in research that evaluates measurement properties of commonly used measures in child and family focused intervention research, for example trialists could consider incorporating the testing of measurement properties into pilot and feasibility studies. Further research is important to ensure that credible evidence can be generated from publicly funded trials and to prevent unnecessary burden on participants from the use of measures that are not reliable.

## Conclusion

There is a dearth of valid and reliable parent report measures of social, behavioural, and emotional outcomes in infants, despite the importance of such outcomes for children’s longer-term prospects. While the SDQ has good measurement properties in school age children, it is not recommended for use with 1-year-olds. A lack of robust measures poses significant challenges to robust evaluation of interventions designed to improve early childhood development.

## Electronic supplementary material

Below is the link to the electronic supplementary material.

Supplementary Material 1

## Data Availability

The E-SEE data sharing plan follows a controlled access model as described in Good Practice Principles for Sharing Individual Participant Data from Publicly Funded Clinical Trials. Anonymised data are available upon request via the ‘Research Data York’ data repository: 10.15124/41fd35ba-bd9c-4c2f-bb50-c8c92b8661ee. To request access to this dataset please email Research Data York repository at lib-open-research@york.ac.uk. Sharing of this quantitative data set will be subject to the completion of a data access request form and, if approved, subject to a data sharing agreement, due to: a) Data containing potentially sensitive participant information such as mental health and domestic violence. b) Ethical concerns around using the data in a way that is not consistent with the PIS, e.g. for research that does not have ethical approval. Data requests will be reviewed by a data access committee, which will include members of the trial management team and independent members from ARC-YH Best Start Steering Group. A data sharing agreement will be required to ensure data is used in accordance with the trial funder, and ethical guidelines.

## Abbreviations

E-SEE: Enhancing Social-Emotional Health and Wellbeing in the Early Years Study
SDQ: Strengths and Difficulties Questionnaire
CFA: Confirmatory Factor Analysis

## References

[1] Black M, Walker S, Fernald L, Andersen C, DiGirolamo A, Lu C, McCoy D, Fink G, Shawar Y, Shiffman J, Devercelli A, Wodon Q, Vargas-Baron E, Grantham-mcgregor S (2017) Early childhood development coming of age: science through the life course. The Lancet 389:77–90 10.1016/S0140-6736(16)31389-7 PMC5884058 27717614

[2] Schroeder C, Gordon B (2020) Assessment and treatment of childhood problems: a clinician’s guide. Guilford press, Guilford

[3] Patel J, Smith R, O’Farrelly C, Iles J, Rosan C, Ryan R, Ramchandani P (2021) Assessing behavior in children aged 12–24 months using the Strengths and Difficulties Questionnaire. Infancy 26(5):724–734 34288359 10.1111/infa.12425

[4] Gridley N, Blower S, Dunn A, Bywater T, Bryant M (2019) Psychometric properties of child (0–5 Years) outcome measures as used in randomized controlled trials of parent programs: a systematic review. Clin Child Fam Psychol Rev 22:388–405 30806864 10.1007/s10567-019-00277-1 PMC6669186

[5] Halle T, Darling-Churchill K (2016) Review of measures of social and emotional development. J Appl Dev Psychol 45:8–18

[6] Pontoppidan M, Niss N, Pejtersen J, Julian M, Væver M (2017) Parent report measures of infant and toddler social-emotional development: a systematic review. Fam Pract 34:127–137 28158522 10.1093/fampra/cmx003

[7] Campbell S, Denham S, Howarth G, Jones S, Whittaker J, Williford A, Willoughby M, Yudron M, Darling-Churchill K (2016) Commentary on the review of measures of early childhood social and emotional development: conceptualization, critique, and recommendations. J Appl Dev Psychol 45:19–41

[8] Goodman R (1997) The Strengths and Difficulties Questionnaire: a research note. J Child Psychol Psychiatry 38:581–586 9255702 10.1111/j.1469-7610.1997.tb01545.x

[9] Kersten P, Czuba K, McPherson K, Dudley M, Elder H, Tauroa R, Vandal A (2016) A systematic review of evidence for the psychometric properties of the Strengths and Difficulties Questionnaire. Int J Behav Dev 40:64–75

[10] Bywater T, Berry V, Blower S, Bursnall M, Cox E, Mason-Jones A, McGilloway S, McKendrick K, Mitchell S, Pickett K, Richardson G, Solaiman K, Teare MD, Walker S, Whittaker K (2022) A randomized controlled trial of a proportionate universal parenting program delivery model (E-SEE Steps) to enhance child social-emotional wellbeing. Plos One. 10.1371/journal.pone.0265200 10.1371/journal.pone.0265200 PMC8979462 35377882

[11] Stone L, Otten R, Engels R, Vermulst A, Janssens J (2010) Psychometric properties of the parent and teacher versions of the strengths and difficulties questionnaire for 4-to 12-year-olds: a review. Clin Child Fam Psychol Rev 13:254–274 20589428 10.1007/s10567-010-0071-2 PMC2919684

[12] Croft S, Stride C, Maughan B, Rowe R (2015) Validity of the strengths and difficulties questionnaire in preschool-aged children. Pediatrics. 10.1542/peds.2014-2920 10.1542/peds.2014-2920 25847804

[13] Goodman R (2001) Psychometric properties of the strengths and difficulties questionnaire. J Am Acad Child Adolesc Psychiatry 40:1337–1345. 10.1097/00004583-200111000-00015 11699809 10.1097/00004583-200111000-00015

[14] D’Souza S, Waldie KE, Peterson ER, Underwood L, Morton SM (2017) Psychometric properties and normative data for the preschool strengths and difficulties questionnaire in two-year-old children. J Abnorm Child Psychol 45(2):345–357 27334707 10.1007/s10802-016-0176-2

[15] Klein AM, Otto Y, Fuchs S, Zenger M, von Klitzing K (2013) Psychometric properties of the parent-rated SDQ in preschoolers. Eur J Psychol Assess 29(2):96–104

[16] Goodman A, Lamping D, Ploubidis G (2010) When to use broader internalising and externalising subscales instead of the hypothesised five subscales on the Strengths and Difficulties Questionnaire (SDQ): data from British parents, teachers and children. J Abnorm Child Psychol 38:1179–1191 20623175 10.1007/s10802-010-9434-x

[17] Bywater T, Berry V, Blower SL, Cohen J, Gridley N, Kiernan K, Mandefield L, Mason-Jones A, McGilloway S, McKendrick K, Pickett K (2018) Enhancing Social-Emotional Health and Wellbeing in the Early Years (E-SEE): a study protocol of a community-based randomised controlled trial with process and economic evaluations of the incredible years infant and toddler parenting programmes, delivered in a proportionate universal model. BMJ open. 10.1136/bmjopen-2018-026906 10.1136/bmjopen-2018-026906 PMC6303737 30573493

[18] Blower SL, Berry V, Bursnall M, Cohen J, Gridley N, Loban A, Mandefield L, Mason-Jones A, McGilloway S, McKendrick K, Mitchell S, Pickett K, Richardson G, Teare MD, Tracey L, Walker S, Whittaker K, Wright J, Bywater T (2021) Enhancing Social-Emotional Outcomes in Early Years (E-SEE): randomized Pilot Study of Incredible Years Infant and Toddler Programs. J Child Fam Stud 30:1933–1949. 10.1007/s10826-021-01991-7

[19] StataCorp (2023) Stata Statistical Software: release 18. StataCorp LLC, College Station, TX

[20] Posit team (2025). RStudio: integrated Development Environment for R. Posit Software, PBC, Boston, MA. URL http://www.posit.co/.

[21] Rosseel Y (2012) lavaan: an R package for structural equation modeling. J Stat Softw 48:1–36

[22] E-SEE Trial Statistical Analysis Plan https://www.york.ac.uk/healthsciences/research/public-health/projects/e-see-trial/. Accessed 20 May 2013.

[23] Hooper D, Coughlan J, Mullen M (2008) Evaluating model fit: a synthesis of the structural equation modelling literature. 7th European Conference on Research Methodology for Business and Management Studies.

[24] Squires J, Bricker D, Twombly E (2002) Ages and stages questionnaires: social-emotional. Brookes Publishing

[25] Terwee CB, Prinsen C, Chiarotto A, De Vet H, Bouter LM, Alonso J, Westerman MJ, Patrick DL, Mokkink LB (2018) COSMIN methodology for assessing the content validity of PROMs–user manual. Amsterdam:VU University Medical Center, 1159–1170. 10.1007/s11136-018-1829-0 PMC5891557 29550964

