# Supplementary material for: Internal consistency and structural validity of the parent-report preschool (2-4 years) Strengths and Difficulties Questionnaire in 1-year-old children
Source: J Patient Rep Outcomes. 2025 Jun 23;9:72. doi: 10.1186/s41687-025-00905-1 (PMC12185824; doi:10.1186/s41687-025-00905-1)
Supplement: Supplementary file 1 — Supplementary Material 1 [file 41687_2025_905_MOESM1_ESM.docx]

SUPPLEMENTARY MATERIAL

Strengths and Difficulties Questionnare: tests of a 2 factor and 5 factor model

2025-03-11

Table of Contents

This file contains results reporting the measurement properties of the Strengths and Difficulties Questionnaire (SDQ) using the E-SEE trial data. We test the fit of a two factor model and a five factor model using Confirmatory Factor Analysis.

# Descriptives

## SDQ item descriptives

### Frequency distributions

**Item wording** 1 Considerate of other people’s feelings, 2 Restless, overactive, cannot stay still for long, 3 Often complains of headaches, stomach-aches or sickness, 4 Shares readily with other children (treats, toys, pencils etc.), 5 Often has temper tantrums or hot tempers, 6 Rather solitary, tends to play alone, 7 Generally obedient, usually does what adults request, 8 Many worries, often seems worried, 9 Helpful if someone is hurt, upset or feeling ill, 10 Constantly fidgeting or squirming, 11 Has at least one good friend, 12 Often fights with other children or bullies them, 13 Often unhappy, down-hearted or tearful, 14 Generally liked by other children, 15 Easily distracted, concentration wanders, 16 Nervous or clingy in new situations, easily loses confidence, 17 Kind to younger children, 18 Often argumentative with adults, 19 Picked on or bullied by other children, 20 Often volunteers to help others (parents, teachers, other children), 21 Can stop and think things out before acting, 22 Can be spiteful to others, 23 Gets on better with adults than with other children, 24 Many fears, easily scared, 25 Sees tasks through to the end, good attention span.

items <- esee %>%
 dplyr::select(sdq_q1:sdq_q25)


library(sjPlot)
tab_stackfrq(items, alternate.rows = TRUE,
 show.n = TRUE, show.na = TRUE)

|  | **Not True** | **Somewhat True** | **Certainly True** | **NA** |
| --- | --- | --- | --- | --- |
| 1. *Considerate* | 46 (9.11 %) | 307 (60.79 %) | 151 (29.90 %) | 1 (0.20 %) |
| 1. *Restless* | 183 (36.24 %) | 227 (44.95 %) | 95 (18.81 %) | 0 (0.00 %) |
| 1. *Complains* | 485 (96.04 %) | 14 (2.77 %) | 2 (0.40 %) | 4 (0.79 %) |
| 1. *Shares* | 39 (7.72 %) | 299 (59.21 %) | 166 (32.87 %) | 1 (0.20 %) |
| 1. *Tantrums* | 192 (38.02 %) | 250 (49.50 %) | 62 (12.28 %) | 1 (0.20 %) |
| 1. *Solitary* | 333 (65.94 %) | 144 (28.51 %) | 27 (5.35 %) | 1 (0.20 %) |
| 1. *Obedient* | 165 (32.67 %) | 309 (61.19 %) | 31 (6.14 %) | 0 (0.00 %) |
| 1. *Worries* | 491 (97.23 %) | 9 (1.78 %) | 2 (0.40 %) | 3 (0.59 %) |
| 1. *Fidgeting* | 309 (61.19 %) | 152 (30.10 %) | 44 (8.71 %) | 0 (0.00 %) |
| 1. *Friend* | 188 (37.23 %) | 216 (42.77 %) | 94 (18.61 %) | 7 (1.39 %) |
| 1. *Fights* | 459 (90.89 %) | 38 (7.52 %) | 7 (1.39 %) | 1 (0.20 %) |
| 1. *Unhappy* | 490 (97.03 %) | 11 (2.18 %) | 3 (0.59 %) | 1 (0.20 %) |
| 1. *Liked* | 343 (67.92 %) | 154 (30.50 %) | 7 (1.39 %) | 1 (0.20 %) |
| 1. *Distracted* | 175 (34.65 %) | 264 (52.28 %) | 64 (12.67 %) | 2 (0.40 %) |
| 1. *Nervous* | 259 (51.29 %) | 198 (39.21 %) | 47 (9.31 %) | 1 (0.20 %) |
| 1. *Kind* | 29 (5.74 %) | 205 (40.59 %) | 264 (52.28 %) | 7 (1.39 %) |
| 1. *Argumentative* | 415 (82.18 %) | 73 (14.46 %) | 9 (1.78 %) | 8 (1.58 %) |
| 1. *Bullied* | 483 (95.64 %) | 12 (2.38 %) | 2 (0.40 %) | 8 (1.58 %) |
| 1. *Volunteers* | 160 (31.68 %) | 223 (44.16 %) | 106 (20.99 %) | 16 (3.17 %) |
| 1. *Thinks* | 41 (8.12 %) | 233 (46.14 %) | 215 (42.57 %) | 16 (3.17 %) |
| 1. *Spiteful* | 433 (85.74 %) | 46 (9.11 %) | 11 (2.18 %) | 15 (2.97 %) |
| 1. *Adults* | 320 (63.37 %) | 153 (30.30 %) | 24 (4.75 %) | 8 (1.58 %) |
| 1. *Fears* | 425 (84.16 %) | 66 (13.07 %) | 10 (1.98 %) | 4 (0.79 %) |
| 1. *Attention* | 71 (14.06 %) | 269 (53.27 %) | 152 (30.10 %) | 13 (2.57 % |

### Means

items %>%
 rstatix::get_summary_stats(,
 type = "common") %>%
 flextable::flextable() %>%
 flextable::autofit()

| variable | n | min | max | median | iqr | mean | sd | se | ci |
| --- | --- | --- | --- | --- | --- | --- | --- | --- | --- |
| sdq_q1 | 504 | 0 | 2 | 1 | 1 | 1.208 | 0.590 | 0.026 | 0.052 |
| sdq_q2 | 505 | 0 | 2 | 1 | 1 | 0.826 | 0.722 | 0.032 | 0.063 |
| sdq_q3 | 501 | 0 | 2 | 0 | 0 | 0.036 | 0.207 | 0.009 | 0.018 |
| sdq_q4 | 504 | 0 | 2 | 1 | 1 | 1.252 | 0.586 | 0.026 | 0.051 |
| sdq_q5 | 504 | 0 | 2 | 1 | 1 | 0.742 | 0.662 | 0.029 | 0.058 |
| sdq_q6 | 504 | 0 | 2 | 0 | 1 | 0.393 | 0.589 | 0.026 | 0.052 |
| sdq_q7 | 505 | 0 | 2 | 1 | 1 | 0.735 | 0.564 | 0.025 | 0.049 |
| sdq_q8 | 502 | 0 | 2 | 0 | 0 | 0.026 | 0.182 | 0.008 | 0.016 |
| sdq_q10 | 505 | 0 | 2 | 0 | 1 | 0.475 | 0.652 | 0.029 | 0.057 |
| sdq_q11 | 498 | 0 | 2 | 1 | 1 | 0.811 | 0.729 | 0.033 | 0.064 |
| sdq_q12 | 504 | 0 | 2 | 0 | 0 | 0.103 | 0.347 | 0.015 | 0.030 |
| sdq_q13 | 504 | 0 | 2 | 0 | 0 | 0.034 | 0.211 | 0.009 | 0.018 |
| sdq_q14 | 504 | 0 | 2 | 0 | 1 | 0.333 | 0.500 | 0.022 | 0.044 |
| sdq_q15 | 503 | 0 | 2 | 1 | 1 | 0.779 | 0.654 | 0.029 | 0.057 |
| sdq_q16 | 504 | 0 | 2 | 0 | 1 | 0.579 | 0.657 | 0.029 | 0.057 |
| sdq_q17 | 498 | 0 | 2 | 2 | 1 | 1.472 | 0.605 | 0.027 | 0.053 |
| sdq_q18 | 497 | 0 | 2 | 0 | 0 | 0.183 | 0.431 | 0.019 | 0.038 |
| sdq_q19 | 497 | 0 | 2 | 0 | 0 | 0.032 | 0.198 | 0.009 | 0.017 |
| sdq_q20 | 489 | 0 | 2 | 1 | 1 | 0.890 | 0.730 | 0.033 | 0.065 |
| sdq_q21 | 489 | 0 | 2 | 1 | 1 | 1.356 | 0.631 | 0.029 | 0.056 |
| sdq_q22 | 490 | 0 | 2 | 0 | 0 | 0.139 | 0.406 | 0.018 | 0.036 |
| sdq_q23 | 497 | 0 | 2 | 0 | 1 | 0.404 | 0.581 | 0.026 | 0.051 |
| sdq_q24 | 501 | 0 | 2 | 0 | 0 | 0.172 | 0.427 | 0.019 | 0.037 |
| sdq_q25 | 492 | 0 | 2 | 1 | 1 | 1.165 | 0.653 | 0.029 | 0.058 |

### Correlations

Correlations are run on complete items only.

head(corr)

## sdq_q1 sdq_q2 sdq_q3 sdq_q4 sdq_q5 sdq_q6 sdq_q7 sdq_q8 sdq_q10 sdq_q11
## sdq_q1 1.00 -0.15 0.03 0.20 -0.20 -0.09 -0.30 0.08 -0.11 -0.26
## sdq_q2 -0.15 1.00 0.01 -0.10 0.34 0.19 0.24 -0.01 0.49 0.11
## sdq_q3 0.03 0.01 1.00 0.09 0.16 0.07 0.02 0.31 0.07 -0.04
## sdq_q4 0.20 -0.10 0.09 1.00 -0.04 -0.13 -0.21 0.05 -0.10 -0.23
## sdq_q5 -0.20 0.34 0.16 -0.04 1.00 0.08 0.24 0.06 0.30 0.03
## sdq_q6 -0.09 0.19 0.07 -0.13 0.08 1.00 0.08 0.12 0.23 -0.04
## sdq_q12 sdq_q13 sdq_q14 sdq_q15 sdq_q16 sdq_q17 sdq_q18 sdq_q19 sdq_q20
## sdq_q1 -0.07 0.02 -0.19 -0.10 -0.06 0.34 0.00 0.03 0.28
## sdq_q2 0.15 0.14 0.12 0.39 0.12 -0.13 0.14 0.08 -0.14
## sdq_q3 0.07 0.24 0.04 0.04 0.04 0.03 0.16 -0.03 0.04
## sdq_q4 -0.08 0.01 -0.32 -0.15 -0.06 0.25 -0.01 0.08 0.20
## sdq_q5 0.18 0.18 0.08 0.30 0.22 -0.04 0.27 0.09 -0.05
## sdq_q6 0.02 0.13 0.11 0.24 0.25 -0.02 0.06 0.06 -0.09
## sdq_q21 sdq_q22 sdq_q23 sdq_q24 sdq_q25
## sdq_q1 -0.31 -0.07 0.03 -0.02 -0.25
## sdq_q2 0.18 0.15 0.08 0.06 0.27
## sdq_q3 -0.05 0.15 0.07 0.03 -0.04
## sdq_q4 -0.23 -0.08 0.00 0.02 -0.24
## sdq_q5 0.13 0.24 0.04 0.09 0.12
## sdq_q6 0.05 0.18 0.23 0.13 0.12

# Plot
ggcorrplot(corr, hc.order = FALSE,
 type = "lower",
 lab = FALSE,
 lab_size = 2,
 insig = "blank",
 method="square",
 colors = c("firebrick", "white", "darkolivegreen4"),
 title="Correlogram of SDQ",
 ggtheme=theme_bw)


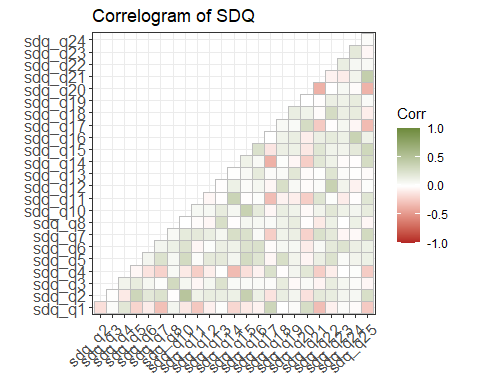


## SDQ total difficulties

### Overall score

This describes the SDQ total difficulties score for the entire sample. As seen below, the sample size is 505, and the mean SDQ score is 9.327 (SD=4.263).

A SDQ total difficulties score was calculated if the child had at least 3 out of the 4 subscales had scores. Hence, there is missing data on individual items as shown above, but SDQ total difficulties scores were calculated for 505 children.

#Summary statistics for total score
#rstatix summary stats numerical
esee %>%
 rstatix::get_summary_stats(sdq_total_diff_score,
 type = "common") %>%
 flextable::flextable() %>%
 flextable::autofit()

| variable | n | min | max | median | iqr | mean | sd | se | ci |
| --- | --- | --- | --- | --- | --- | --- | --- | --- | --- |
| sdq_total_diff_score | 505 | 0 | 25 | 9 | 6 | 9.327 | 4.263 | 0.19 | 0.373 |

sdq <- ggplot(esee, aes(x=sdq_total_diff_score)) +
 geom_density(lwd=1,
 alpha=0.7,
 fill="tan1")

sdq + theme_light() + labs(x="SDQ total difficulties score", y="Density")


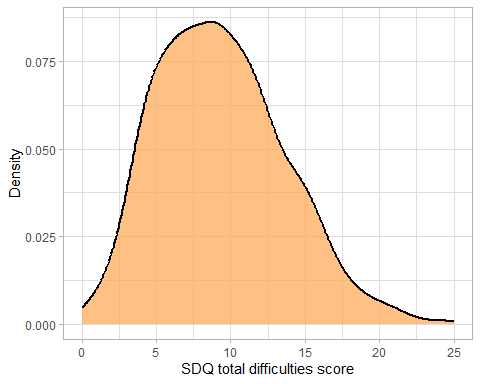


### Scores by treatment group

As this is a trial data set, we have provided summaries of the SDQ total difficulties score by the treatment group (intervention vs control).

#The first (larger) group are intervention, and we label them as such below:
treatment <- factor(esee$rand_randomisation_grp, labels = c("intervention", "control"))


# Density ridge plot
sdq_t2 <- ggplot(esee, aes(x = sdq_total_diff_score, y=treatment, fill = treatment)) +
 geom_density_ridges(lwd=1,
 alpha=0.7
 )
sdq_t2 + theme_light() + labs(x="SDQ total difficulties score", y="Density")


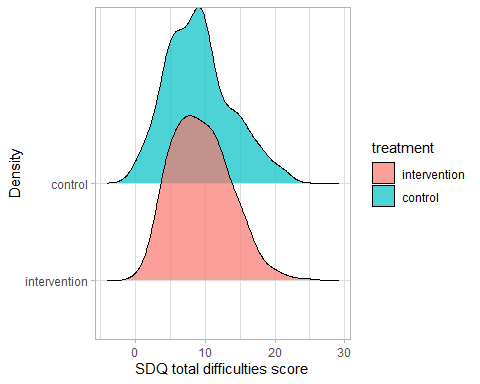


### Scores by age in months

As this study tests the validity of the SDQ in a group of young children, we present a density plot of SDQ scores by age in weeks (77-91 weeks).

**Due to risk of reidentification, we do not present tabs of SDQ scores by age. Instead, we present a density plot by age in months.**

#Density plot
sdq_t <- ggplot(temp, aes(sdq_total_diff_score, stat(count), colour=age, fill = ageinweeks)) +
 geom_density(lwd=1,
 alpha=0.7,
 position="stack"
 )
#sdq_t + theme_light() + labs(x="SDQ total difficulties score", y="Density")

# Density ridge plot
sdq_age <- ggplot(temp, aes(x = sdq_total_diff_score, y=age, fill = age)) +
 geom_density_ridges(lwd=1,
 alpha=0.7
 )
sdq_age + theme_light() + labs(x="SDQ total difficulties score", y="Density")


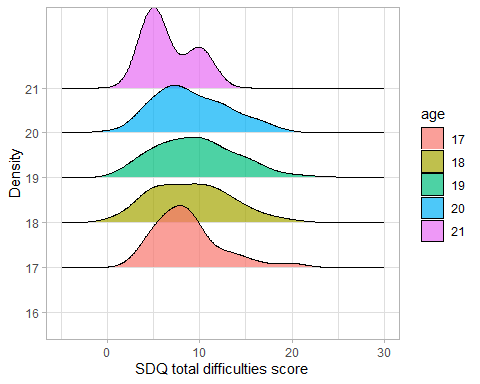


# Two factor model

First we test the 2 factor model of the SDQ, which has an ‘internalising’ and an ‘externalising’ scale, and looks like below:

#2 factor model
m1b <- ' internalising =~ sdq_q23 + sdq_q19 + sdq_q14 + sdq_q11 + sdq_q6 + sdq_q24 + sdq_q16 + sdq_q13 + sdq_q8 + sdq_q3
 externalising =~ sdq_q18 + sdq_q12 + sdq_q7 + sdq_q5 + sdq_q25 + sdq_q21 + sdq_q15 + sdq_q10 + sdq_q2'

factor2model <- cfa(m1b, data=esee, mimic =c("MPlus"), estimator = "MLR")

semPaths(factor2model,
 layout = "tree2", rotation = 2,
 intercepts = FALSE, residuals=FALSE,
 sizeMan=6, sizeMan2=2,
 width=10, height=10,
 )


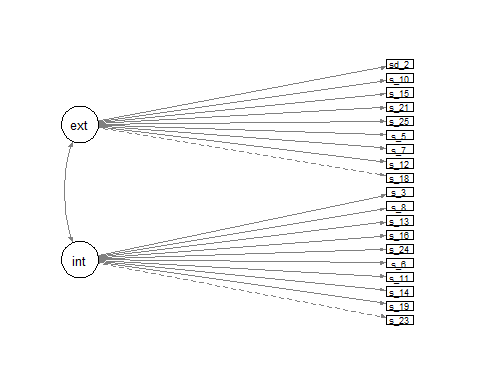


## Internal consistency of subscales

### Internalising

# Omega: internal consistency
internalising <- esee %>%
 dplyr::select(sdq_q23, sdq_q19, sdq_q14, sdq_q11, sdq_q6, sdq_q24, sdq_q16, sdq_q13, sdq_q8, sdq_q3)

summary(omega(internalising, plot = F))

## Omega
## omega(m = internalising, plot = F)
## Alpha: 0.47
## G.6: 0.5
## Omega Hierarchical: 0.36
## Omega H asymptotic: 0.63
## Omega Total 0.57
##
## With eigenvalues of:
## g F1* F2* F3*
## 0.981 0.071 0.760 0.703
## The degrees of freedom for the model is 18 and the fit was 0.13
## The number of observations was 505 with Chi Square = 62.34 with prob < 0
##
## The root mean square of the residuals is 0.04
## The df corrected root mean square of the residuals is 0.08
##
## RMSEA and the 0.9 confidence intervals are 0.07 0.051 0.089
## BIC = -49.7Explained Common Variance of the general factor = 0.39
##
## Total, General and Subset omega for each subset
## g F1* F2* F3*
## Omega total for total scores and subscales 0.57 0.49 0.51 0.13
## Omega general for total scores and subscales 0.36 0.45 0.09 0.11
## Omega group for total scores and subscales 0.16 0.04 0.42 0.01

### Externalising

# Omega: internal consistency
externalising <- esee %>%
 dplyr::select(sdq_q18, sdq_q12, sdq_q7, sdq_q5, sdq_q25, sdq_q21, sdq_q15, sdq_q10, sdq_q2)

summary(omega(externalising, plot = F))

## Omega
## omega(m = externalising, plot = F)
## Alpha: 0.7
## G.6: 0.71
## Omega Hierarchical: 0.52
## Omega H asymptotic: 0.68
## Omega Total 0.76
##
## With eigenvalues of:
## g F1* F2* F3*
## 1.78 0.00 0.99 0.65
## The degrees of freedom for the model is 12 and the fit was 0.02
## The number of observations was 505 with Chi Square = 8.35 with prob < 0.76
##
## The root mean square of the residuals is 0.02
## The df corrected root mean square of the residuals is 0.05
##
## RMSEA and the 0.9 confidence intervals are 0 0 0.032
## BIC = -66.35Explained Common Variance of the general factor = 0.52
##
## Total, General and Subset omega for each subset
## g F1* F2* F3*
## Omega total for total scores and subscales 0.76 NA 0.74 0.53
## Omega general for total scores and subscales 0.52 NA 0.51 0.15
## Omega group for total scores and subscales 0.22 NA 0.24 0.38

## Model output

summary(factor2model, standardized=TRUE, fit.measures=TRUE)

## lavaan 0.6.16 ended normally after 123 iterations
##
## Estimator ML
## Optimization method NLMINB
## Number of model parameters 58
##
## Number of observations 505
## Number of missing patterns 23
##
## Model Test User Model:
## Standard Scaled
## Test Statistic 626.067 482.018
## Degrees of freedom 151 151
## P-value (Chi-square) 0.000 0.000
## Scaling correction factor 1.299
## Yuan-Bentler correction (Mplus variant)
##
## Model Test Baseline Model:
##
## Test statistic 1395.379 1045.905
## Degrees of freedom 171 171
## P-value 0.000 0.000
## Scaling correction factor 1.334
##
## User Model versus Baseline Model:
##
## Comparative Fit Index (CFI) 0.612 0.622
## Tucker-Lewis Index (TLI) 0.561 0.572
##
## Robust Comparative Fit Index (CFI) 0.642
## Robust Tucker-Lewis Index (TLI) 0.595
##
## Loglikelihood and Information Criteria:
##
## Loglikelihood user model (H0) -5717.793 -5717.793
## Scaling correction factor 3.177
## for the MLR correction
## Loglikelihood unrestricted model (H1) NA NA
## Scaling correction factor 1.820
## for the MLR correction
##
## Akaike (AIC) 11551.586 11551.586
## Bayesian (BIC) 11796.610 11796.610
## Sample-size adjusted Bayesian (SABIC) 11612.512 11612.512
##
## Root Mean Square Error of Approximation:
##
## RMSEA 0.079 0.066
## 90 Percent confidence interval - lower 0.073 0.060
## 90 Percent confidence interval - upper 0.085 0.072
## P-value H_0: RMSEA <= 0.050 0.000 0.000
## P-value H_0: RMSEA >= 0.080 0.400 0.000
##
## Robust RMSEA 0.074
## 90 Percent confidence interval - lower 0.065
## 90 Percent confidence interval - upper 0.082
## P-value H_0: Robust RMSEA <= 0.050 0.000
## P-value H_0: Robust RMSEA >= 0.080 0.103
##
## Standardized Root Mean Square Residual:
##
## SRMR 0.077 0.077
##
## Parameter Estimates:
##
## Standard errors Sandwich
## Information bread Observed
## Observed information based on Hessian
##
## Latent Variables:
## Estimate Std.Err z-value P(>|z|) Std.lv Std.all
## internalising =~
## sdq_q23 1.000 0.168 0.290
## sdq_q19 0.356 0.165 2.156 0.031 0.060 0.303
## sdq_q14 0.342 0.253 1.355 0.175 0.058 0.115
## sdq_q11 0.050 0.342 0.146 0.884 0.008 0.012
## sdq_q6 1.518 0.394 3.853 0.000 0.256 0.435
## sdq_q24 1.116 0.364 3.068 0.002 0.188 0.441
## sdq_q16 2.225 0.648 3.432 0.001 0.375 0.572
## sdq_q13 0.378 0.191 1.982 0.047 0.064 0.302
## sdq_q8 0.264 0.162 1.632 0.103 0.044 0.244
## sdq_q3 0.228 0.154 1.481 0.139 0.038 0.186
## externalising =~
## sdq_q18 1.000 0.125 0.289
## sdq_q12 0.564 0.147 3.849 0.000 0.070 0.203
## sdq_q7 1.861 0.503 3.698 0.000 0.232 0.412
## sdq_q5 2.820 0.589 4.790 0.000 0.352 0.532
## sdq_q25 2.180 0.658 3.313 0.001 0.272 0.417
## sdq_q21 1.551 0.525 2.955 0.003 0.193 0.307
## sdq_q15 3.327 0.826 4.028 0.000 0.415 0.635
## sdq_q10 3.210 0.768 4.182 0.000 0.400 0.615
## sdq_q2 3.771 0.911 4.139 0.000 0.470 0.652
##
## Covariances:
## Estimate Std.Err z-value P(>|z|) Std.lv Std.all
## internalising ~~
## externalising 0.010 0.004 2.790 0.005 0.478 0.478
##
## Intercepts:
## Estimate Std.Err z-value P(>|z|) Std.lv Std.all
## .sdq_q23 0.404 0.026 15.520 0.000 0.404 0.696
## .sdq_q19 0.032 0.009 3.628 0.000 0.032 0.164
## .sdq_q14 0.333 0.022 14.969 0.000 0.333 0.667
## .sdq_q11 0.811 0.033 24.845 0.000 0.811 1.114
## .sdq_q6 0.393 0.026 15.003 0.000 0.393 0.669
## .sdq_q24 0.172 0.019 8.999 0.000 0.172 0.402
## .sdq_q16 0.579 0.029 19.832 0.000 0.579 0.883
## .sdq_q13 0.034 0.009 3.590 0.000 0.034 0.161
## .sdq_q8 0.026 0.008 3.189 0.001 0.026 0.143
## .sdq_q3 0.036 0.009 3.882 0.000 0.036 0.176
## .sdq_q18 0.184 0.019 9.474 0.000 0.184 0.426
## .sdq_q12 0.103 0.015 6.679 0.000 0.103 0.298
## .sdq_q7 0.735 0.025 29.290 0.000 0.735 1.303
## .sdq_q5 0.743 0.029 25.200 0.000 0.743 1.123
## .sdq_q25 1.163 0.029 39.573 0.000 1.163 1.782
## .sdq_q21 1.353 0.028 47.508 0.000 1.353 2.148
## .sdq_q15 0.778 0.029 26.744 0.000 0.778 1.192
## .sdq_q10 0.475 0.029 16.408 0.000 0.475 0.730
## .sdq_q2 0.826 0.032 25.730 0.000 0.826 1.145
## internalising 0.000 0.000 0.000
## externalising 0.000 0.000 0.000
##
## Variances:
## Estimate Std.Err z-value P(>|z|) Std.lv Std.all
## .sdq_q23 0.309 0.023 13.493 0.000 0.309 0.916
## .sdq_q19 0.036 0.011 3.301 0.001 0.036 0.908
## .sdq_q14 0.247 0.016 15.779 0.000 0.247 0.987
## .sdq_q11 0.531 0.023 23.264 0.000 0.531 1.000
## .sdq_q6 0.280 0.026 10.881 0.000 0.280 0.811
## .sdq_q24 0.147 0.021 6.943 0.000 0.147 0.806
## .sdq_q16 0.290 0.044 6.613 0.000 0.290 0.673
## .sdq_q13 0.040 0.012 3.334 0.001 0.040 0.909
## .sdq_q8 0.031 0.011 2.739 0.006 0.031 0.940
## .sdq_q3 0.041 0.012 3.529 0.000 0.041 0.965
## .sdq_q18 0.170 0.019 9.072 0.000 0.170 0.916
## .sdq_q12 0.115 0.020 5.905 0.000 0.115 0.959
## .sdq_q7 0.264 0.017 15.339 0.000 0.264 0.831
## .sdq_q5 0.314 0.022 14.283 0.000 0.314 0.717
## .sdq_q25 0.352 0.024 14.363 0.000 0.352 0.827
## .sdq_q21 0.359 0.023 15.404 0.000 0.359 0.906
## .sdq_q15 0.254 0.024 10.497 0.000 0.254 0.597
## .sdq_q10 0.263 0.025 10.534 0.000 0.263 0.622
## .sdq_q2 0.299 0.025 11.836 0.000 0.299 0.575
## internalising 0.028 0.014 2.055 0.040 1.000 1.000
## externalising 0.016 0.007 2.201 0.028 1.000 1.000

### Robust fit output

fit2 <- cfa(factor2model, data = esee)
fit.robust <- c("chisq.robust", "cfi.robust", "rmsea.robust", "aic")

fitMeasures(factor2model, fit.robust)

## cfi.robust rmsea.robust aic
## 0.642 0.074 11551.586

## Modification indices

modindices(factor2model, sort = TRUE, maximum.number = 5)

## lhs op rhs mi epc sepc.lv sepc.all sepc.nox
## 243 sdq_q25 ~~ sdq_q21 57.717 0.129 0.129 0.363 0.363
## 117 sdq_q14 ~~ sdq_q11 55.530 0.121 0.121 0.335 0.335
## 198 sdq_q8 ~~ sdq_q3 36.331 0.010 0.010 0.279 0.279
## 162 sdq_q24 ~~ sdq_q16 31.156 0.077 0.077 0.375 0.375
## 217 sdq_q18 ~~ sdq_q12 25.391 0.032 0.032 0.231 0.231

# Five factor model

Next we test the 5 factor model of SDQ: 1) emotional symptoms (5 items), 2) conduct problems (5 items), 3) hyperactivity/inattention (5 items), 4) peer relationship problems (5 items), and 5) prosocial behaviour (5 items). This model looks like below:

m1a <- ' prosocial =~ sdq_q1 + sdq_q4 + sdq_q9 + sdq_q17 + sdq_q20
 hyperactive =~ sdq_q25 + sdq_q21 + sdq_q15 + sdq_q10 + sdq_q2
 peerproblems =~ sdq_q23 + sdq_q19 + sdq_q14 + sdq_q11 + sdq_q6
 conductproblems =~ sdq_q18 + sdq_q12 + sdq_q7 + sdq_q5
 emotionalsymptoms =~ sdq_q24 + sdq_q16 + sdq_q13 + sdq_q8 + sdq_q3'

factor5model <- cfa(m1a, data=esee, mimic =c("MPlus"), estimator = "MLR")

fit5 <- cfa(factor5model, data = esee)

semPaths(factor5model,
 layout = "tree2", rotation = 2,
 intercepts = FALSE, residuals=FALSE,
 sizeMan=6, sizeMan2=2,
 width=10, height=10,
 )


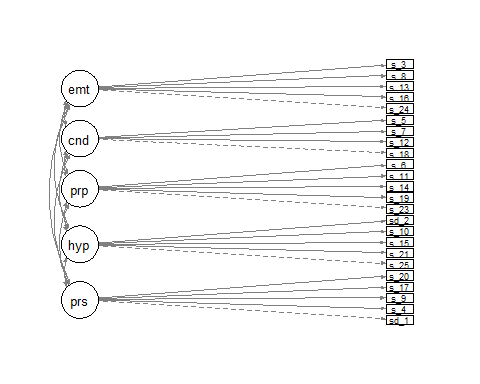


## Internal consistency of subscales

### Prosocial

prosocial <- esee %>%
 dplyr::select(sdq_q1, sdq_q4, sdq_q9, sdq_q17, sdq_q20)

summary(omega(prosocial, plot = F))

## Omega
## omega(m = prosocial, plot = F)
## Alpha: 0.68
## G.6: 0.64
## Omega Hierarchical: 0.65
## Omega H asymptotic: 0.92
## Omega Total 0.71
##
## With eigenvalues of:
## g F1* F2* F3*
## 1.504 0.000 0.213 0.092
## The degrees of freedom for the model is -2 and the fit was 0
## The number of observations was 505 with Chi Square = 0 with prob < NA
##
## The root mean square of the residuals is 0
## The df corrected root mean square of the residuals is NA
## Explained Common Variance of the general factor = 0.83
##
## Total, General and Subset omega for each subset
## g F1* F2* F3*
## Omega total for total scores and subscales 0.71 NA 0.43 0.65
## Omega general for total scores and subscales 0.65 NA 0.28 0.65
## Omega group for total scores and subscales 0.04 NA 0.15 0.01

### Hyperactive

hyperactive <- esee %>%
 dplyr::select(sdq_q25, sdq_q21, sdq_q15, sdq_q10, sdq_q2)

summary(omega(hyperactive, plot = F))

## Omega
## omega(m = hyperactive, plot = F)
## Alpha: 0.67
## G.6: 0.66
## Omega Hierarchical: 0.43
## Omega H asymptotic: 0.57
## Omega Total 0.75
##
## With eigenvalues of:
## g F1* F2* F3*
## 0.972 0.723 0.568 0.032
## The degrees of freedom for the model is -2 and the fit was 0
## The number of observations was 505 with Chi Square = 0 with prob < NA
##
## The root mean square of the residuals is 0
## The df corrected root mean square of the residuals is NA
## Explained Common Variance of the general factor = 0.42
##
## Total, General and Subset omega for each subset
## g F1* F2* F3*
## Omega total for total scores and subscales 0.75 0.71 0.60 NA
## Omega general for total scores and subscales 0.43 0.35 0.21 NA
## Omega group for total scores and subscales 0.28 0.36 0.39 NA

### Peer problems

peerproblems <- esee %>%
 dplyr::select(sdq_q23, sdq_q19, sdq_q14, sdq_q11, sdq_q6)

summary(omega(peerproblems, plot = F))

## Omega
## omega(m = peerproblems, plot = F)
## Alpha: 0.16
## G.6: 0.2
## Omega Hierarchical: 0.31
## Omega H asymptotic: 0.82
## Omega Total 0.37
##
## With eigenvalues of:
## g F1* F2* F3*
## 0.56 0.69 0.00 0.16
## The degrees of freedom for the model is -2 and the fit was 0
## The number of observations was 505 with Chi Square = 0 with prob < NA
##
## The root mean square of the residuals is 0.02
## The df corrected root mean square of the residuals is NA
## Explained Common Variance of the general factor = 0.4
##
## Total, General and Subset omega for each subset
## g F1* F2* F3*
## Omega total for total scores and subscales 0.37 0.05 NA 0.45
## Omega general for total scores and subscales 0.31 0.05 NA 0.39
## Omega group for total scores and subscales 0.03 0.00 NA 0.06

### Conduct problems

conduct <- esee %>%
 dplyr::select(sdq_q18, sdq_q12, sdq_q7, sdq_q5)

summary(omega(conduct, plot = F))

## Omega
## omega(m = conduct, plot = F)
## Alpha: 0.5
## G.6: 0.44
## Omega Hierarchical: 0.44
## Omega H asymptotic: 0.78
## Omega Total 0.56
##
## With eigenvalues of:
## g F1* F2* F3*
## 0.762 0.082 0.284 0.070
## The degrees of freedom for the model is -3 and the fit was 0
## The number of observations was 505 with Chi Square = 0 with prob < NA
##
## The root mean square of the residuals is 0
## The df corrected root mean square of the residuals is NA
## Explained Common Variance of the general factor = 0.64
##
## Total, General and Subset omega for each subset
## g F1* F2* F3*
## Omega total for total scores and subscales 0.56 0.45 0.42 NA
## Omega general for total scores and subscales 0.44 0.39 0.19 NA
## Omega group for total scores and subscales 0.11 0.06 0.23 NA

### Emotional symptoms

emotional <- esee %>%
 dplyr::select(sdq_q24, sdq_q16, sdq_q13, sdq_q8, sdq_q3)

summary(omega(emotional, plot = F))

## Omega
## omega(m = emotional, plot = F)
## Alpha: 0.47
## G.6: 0.46
## Omega Hierarchical: 0.32
## Omega H asymptotic: 0.52
## Omega Total 0.62
##
## With eigenvalues of:
## g F1* F2* F3*
## 0.78 0.41 0.74 0.00
## The degrees of freedom for the model is -2 and the fit was 0
## The number of observations was 505 with Chi Square = 0 with prob < NA
##
## The root mean square of the residuals is 0.02
## The df corrected root mean square of the residuals is NA
## Explained Common Variance of the general factor = 0.41
##
## Total, General and Subset omega for each subset
## g F1* F2* F3*
## Omega total for total scores and subscales 0.62 0.63 0.51 NA
## Omega general for total scores and subscales 0.32 0.46 0.05 NA
## Omega group for total scores and subscales 0.32 0.17 0.46 NA

## Model output

summary(factor5model, standardized=TRUE, fit.measures=TRUE)

## lavaan 0.6.16 ended normally after 202 iterations
##
## Estimator ML
## Optimization method NLMINB
## Number of model parameters 82
##
## Number of observations 505
## Number of missing patterns 30
##
## Model Test User Model:
## Standard Scaled
## Test Statistic 836.813 710.734
## Degrees of freedom 242 242
## P-value (Chi-square) 0.000 0.000
## Scaling correction factor 1.177
## Yuan-Bentler correction (Mplus variant)
##
## Model Test Baseline Model:
##
## Test statistic 2113.435 1705.546
## Degrees of freedom 276 276
## P-value 0.000 0.000
## Scaling correction factor 1.239
##
## User Model versus Baseline Model:
##
## Comparative Fit Index (CFI) 0.676 0.672
## Tucker-Lewis Index (TLI) 0.631 0.626
##
## Robust Comparative Fit Index (CFI) 0.706
## Robust Tucker-Lewis Index (TLI) 0.665
##
## Loglikelihood and Information Criteria:
##
## Loglikelihood user model (H0) -7865.298 -7865.298
## Scaling correction factor 2.655
## for the MLR correction
## Loglikelihood unrestricted model (H1) NA NA
## Scaling correction factor 1.551
## for the MLR correction
##
## Akaike (AIC) 15894.596 15894.596
## Bayesian (BIC) 16241.010 16241.010
## Sample-size adjusted Bayesian (SABIC) 15980.733 15980.733
##
## Root Mean Square Error of Approximation:
##
## RMSEA 0.070 0.062
## 90 Percent confidence interval - lower 0.065 0.057
## 90 Percent confidence interval - upper 0.075 0.067
## P-value H_0: RMSEA <= 0.050 0.000 0.000
## P-value H_0: RMSEA >= 0.080 0.001 0.000
##
## Robust RMSEA 0.065
## 90 Percent confidence interval - lower 0.059
## 90 Percent confidence interval - upper 0.072
## P-value H_0: Robust RMSEA <= 0.050 0.000
## P-value H_0: Robust RMSEA >= 0.080 0.000
##
## Standardized Root Mean Square Residual:
##
## SRMR 0.081 0.081
##
## Parameter Estimates:
##
## Standard errors Sandwich
## Information bread Observed
## Observed information based on Hessian
##
## Latent Variables:
## Estimate Std.Err z-value P(>|z|) Std.lv Std.all
## prosocial =~
## sdq_q1 1.000 0.324 0.550
## sdq_q4 0.774 0.125 6.184 0.000 0.251 0.428
## sdq_q9 1.324 0.127 10.444 0.000 0.429 0.640
## sdq_q17 1.125 0.135 8.309 0.000 0.365 0.603
## sdq_q20 1.182 0.144 8.218 0.000 0.383 0.526
## hyperactive =~
## sdq_q25 1.000 0.330 0.506
## sdq_q21 0.754 0.093 8.087 0.000 0.249 0.395
## sdq_q15 1.244 0.200 6.231 0.000 0.410 0.628
## sdq_q10 1.125 0.266 4.233 0.000 0.371 0.570
## sdq_q2 1.394 0.283 4.919 0.000 0.460 0.637
## peerproblems =~
## sdq_q23 1.000 0.052 0.089
## sdq_q19 0.253 0.242 1.048 0.295 0.013 0.066
## sdq_q14 -5.536 4.917 -1.126 0.260 -0.287 -0.573
## sdq_q11 -8.032 6.610 -1.215 0.224 -0.416 -0.571
## sdq_q6 -1.075 1.857 -0.579 0.563 -0.056 -0.095
## conductproblems =~
## sdq_q18 1.000 0.156 0.363
## sdq_q12 0.614 0.171 3.590 0.000 0.096 0.277
## sdq_q7 1.655 0.789 2.096 0.036 0.259 0.459
## sdq_q5 2.681 0.586 4.575 0.000 0.419 0.634
## emotionalsymptoms =~
## sdq_q24 1.000 0.208 0.488
## sdq_q16 2.155 0.861 2.503 0.012 0.449 0.685
## sdq_q13 0.253 0.195 1.296 0.195 0.053 0.250
## sdq_q8 0.172 0.221 0.776 0.438 0.036 0.196
## sdq_q3 0.141 0.241 0.585 0.558 0.029 0.142
##
## Covariances:
## Estimate Std.Err z-value P(>|z|) Std.lv Std.all
## prosocial ~~
## hyperactive -0.052 0.017 -3.020 0.003 -0.482 -0.482
## peerproblems 0.014 0.012 1.163 0.245 0.809 0.809
## conductproblms -0.019 0.006 -3.317 0.001 -0.367 -0.367
## emotinlsymptms -0.006 0.008 -0.732 0.464 -0.085 -0.085
## hyperactive ~~
## peerproblems -0.006 0.005 -1.282 0.200 -0.350 -0.350
## conductproblms 0.039 0.009 4.400 0.000 0.747 0.747
## emotinlsymptms 0.025 0.007 3.507 0.000 0.367 0.367
## peerproblems ~~
## conductproblms -0.002 0.002 -1.287 0.198 -0.267 -0.267
## emotinlsymptms -0.000 0.001 -0.325 0.745 -0.038 -0.038
## conductproblems ~~
## emotinlsymptms 0.013 0.008 1.647 0.099 0.405 0.405
##
## Intercepts:
## Estimate Std.Err z-value P(>|z|) Std.lv Std.all
## .sdq_q1 1.208 0.026 45.998 0.000 1.208 2.048
## .sdq_q4 1.252 0.026 47.961 0.000 1.252 2.136
## .sdq_q9 1.067 0.030 35.656 0.000 1.067 1.591
## .sdq_q17 1.471 0.027 54.372 0.000 1.471 2.433
## .sdq_q20 0.890 0.033 27.128 0.000 0.890 1.223
## .sdq_q25 1.162 0.029 39.580 0.000 1.162 1.782
## .sdq_q21 1.352 0.028 47.470 0.000 1.352 2.147
## .sdq_q15 0.778 0.029 26.737 0.000 0.778 1.191
## .sdq_q10 0.475 0.029 16.408 0.000 0.475 0.730
## .sdq_q2 0.826 0.032 25.730 0.000 0.826 1.145
## .sdq_q23 0.404 0.026 15.519 0.000 0.404 0.696
## .sdq_q19 0.032 0.009 3.623 0.000 0.032 0.163
## .sdq_q14 0.333 0.022 14.970 0.000 0.333 0.667
## .sdq_q11 0.810 0.033 24.898 0.000 0.810 1.113
## .sdq_q6 0.393 0.026 14.998 0.000 0.393 0.668
## .sdq_q18 0.184 0.019 9.469 0.000 0.184 0.426
## .sdq_q12 0.103 0.015 6.679 0.000 0.103 0.298
## .sdq_q7 0.735 0.025 29.290 0.000 0.735 1.303
## .sdq_q5 0.743 0.029 25.200 0.000 0.743 1.123
## .sdq_q24 0.172 0.019 8.974 0.000 0.172 0.403
## .sdq_q16 0.579 0.029 19.835 0.000 0.579 0.883
## .sdq_q13 0.034 0.009 3.586 0.000 0.034 0.160
## .sdq_q8 0.026 0.008 3.181 0.001 0.026 0.143
## .sdq_q3 0.036 0.009 3.854 0.000 0.036 0.175
## prosocial 0.000 0.000 0.000
## hyperactive 0.000 0.000 0.000
## peerproblems 0.000 0.000 0.000
## conductproblms 0.000 0.000 0.000
## emotinlsymptms 0.000 0.000 0.000
##
## Variances:
## Estimate Std.Err z-value P(>|z|) Std.lv Std.all
## .sdq_q1 0.243 0.019 12.810 0.000 0.243 0.698
## .sdq_q4 0.280 0.019 15.047 0.000 0.280 0.817
## .sdq_q9 0.266 0.023 11.540 0.000 0.266 0.591
## .sdq_q17 0.233 0.022 10.480 0.000 0.233 0.636
## .sdq_q20 0.383 0.027 13.976 0.000 0.383 0.723
## .sdq_q25 0.317 0.031 10.103 0.000 0.317 0.744
## .sdq_q21 0.335 0.029 11.721 0.000 0.335 0.844
## .sdq_q15 0.258 0.024 10.554 0.000 0.258 0.605
## .sdq_q10 0.286 0.031 9.097 0.000 0.286 0.675
## .sdq_q2 0.309 0.034 9.219 0.000 0.309 0.594
## .sdq_q23 0.335 0.023 14.725 0.000 0.335 0.992
## .sdq_q19 0.039 0.012 3.123 0.002 0.039 0.996
## .sdq_q14 0.168 0.019 8.671 0.000 0.168 0.671
## .sdq_q11 0.357 0.037 9.660 0.000 0.357 0.674
## .sdq_q6 0.343 0.024 14.395 0.000 0.343 0.991
## .sdq_q18 0.161 0.020 7.975 0.000 0.161 0.868
## .sdq_q12 0.111 0.018 6.021 0.000 0.111 0.923
## .sdq_q7 0.251 0.030 8.306 0.000 0.251 0.789
## .sdq_q5 0.262 0.047 5.577 0.000 0.262 0.599
## .sdq_q24 0.139 0.020 7.071 0.000 0.139 0.761
## .sdq_q16 0.228 0.157 1.458 0.145 0.228 0.531
## .sdq_q13 0.042 0.013 3.271 0.001 0.042 0.937
## .sdq_q8 0.032 0.012 2.724 0.006 0.032 0.961
## .sdq_q3 0.042 0.012 3.539 0.000 0.042 0.980
## prosocial 0.105 0.019 5.481 0.000 1.000 1.000
## hyperactive 0.109 0.031 3.518 0.000 1.000 1.000
## peerproblems 0.003 0.005 0.575 0.565 1.000 1.000
## conductproblms 0.024 0.014 1.722 0.085 1.000 1.000
## emotinlsymptms 0.043 0.015 2.852 0.004 1.000 1.000

### Robust fit output

fit.robust <- c("chisq.robust", "cfi.robust", "rmsea.robust", "aic")

fitMeasures(factor5model, fit.robust)

## cfi.robust rmsea.robust aic
## 0.706 0.065 15894.596

## Modification indices

modindices(factor5model, sort = TRUE, maximum.number = 10)

## lhs op rhs mi epc sepc.lv sepc.all sepc.nox
## 93 prosocial =~ sdq_q25 58.649 -0.936 -0.303 -0.465 -0.465
## 105 prosocial =~ sdq_q7 58.324 -0.815 -0.264 -0.469 -0.469
## 94 prosocial =~ sdq_q21 57.903 -0.917 -0.297 -0.472 -0.472
## 136 peerproblems =~ sdq_q25 49.606 -5.322 -0.276 -0.422 -0.422
## 143 peerproblems =~ sdq_q7 48.229 -4.681 -0.242 -0.430 -0.430
## 137 peerproblems =~ sdq_q21 44.289 -4.969 -0.257 -0.409 -0.409
## 294 sdq_q25 ~~ sdq_q21 42.168 0.107 0.107 0.327 0.327
## 464 sdq_q8 ~~ sdq_q3 39.276 0.010 0.010 0.287 0.287
## 184 emotionalsymptoms =~ sdq_q6 38.630 1.016 0.212 0.360 0.360
## 345 sdq_q10 ~~ sdq_q2 37.751 0.112 0.112 0.378 0.378

# Three factor model (supplementary analysis)

#2 factor model
m1c <- ' internalising =~ sdq_q23 + sdq_q19 + sdq_q14 + sdq_q11 + sdq_q6 + sdq_q24 + sdq_q16 + sdq_q13 + sdq_q8 + sdq_q3
 externalising =~ sdq_q18 + sdq_q12 + sdq_q7 + sdq_q5 + sdq_q25 + sdq_q21 + sdq_q15 + sdq_q10 + sdq_q2
 prosocial =~ sdq_q1 + sdq_q4 + sdq_q9 + sdq_q17 + sdq_q20'

factor3model <- cfa(m1c, data=esee, mimic =c("MPlus"), estimator = "MLR")

semPaths(factor3model,
 layout = "tree2", rotation = 2,
 intercepts = FALSE, residuals=FALSE,
 sizeMan=6, sizeMan2=2,
 width=10, height=10,
 )


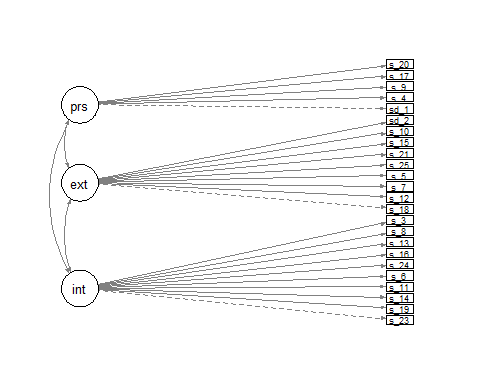


## Internal consistency of subscales

### Internalising

# Omega: internal consistency
internalising <- esee %>%
 dplyr::select(sdq_q23, sdq_q19, sdq_q14, sdq_q11, sdq_q6, sdq_q24, sdq_q16, sdq_q13, sdq_q8, sdq_q3)

summary(omega(internalising, plot = F))

## Omega
## omega(m = internalising, plot = F)
## Alpha: 0.47
## G.6: 0.5
## Omega Hierarchical: 0.36
## Omega H asymptotic: 0.63
## Omega Total 0.57
##
## With eigenvalues of:
## g F1* F2* F3*
## 0.981 0.071 0.760 0.703
## The degrees of freedom for the model is 18 and the fit was 0.13
## The number of observations was 505 with Chi Square = 62.34 with prob < 0
##
## The root mean square of the residuals is 0.04
## The df corrected root mean square of the residuals is 0.08
##
## RMSEA and the 0.9 confidence intervals are 0.07 0.051 0.089
## BIC = -49.7Explained Common Variance of the general factor = 0.39
##
## Total, General and Subset omega for each subset
## g F1* F2* F3*
## Omega total for total scores and subscales 0.57 0.49 0.51 0.13
## Omega general for total scores and subscales 0.36 0.45 0.09 0.11
## Omega group for total scores and subscales 0.16 0.04 0.42 0.01

### Externalising

# Omega: internal consistency
externalising <- esee %>%
 dplyr::select(sdq_q18, sdq_q12, sdq_q7, sdq_q5, sdq_q25, sdq_q21, sdq_q15, sdq_q10, sdq_q2)

summary(omega(externalising, plot = F))

## Omega
## omega(m = externalising, plot = F)
## Alpha: 0.7
## G.6: 0.71
## Omega Hierarchical: 0.52
## Omega H asymptotic: 0.68
## Omega Total 0.76
##
## With eigenvalues of:
## g F1* F2* F3*
## 1.78 0.00 0.99 0.65
## The degrees of freedom for the model is 12 and the fit was 0.02
## The number of observations was 505 with Chi Square = 8.35 with prob < 0.76
##
## The root mean square of the residuals is 0.02
## The df corrected root mean square of the residuals is 0.05
##
## RMSEA and the 0.9 confidence intervals are 0 0 0.032
## BIC = -66.35Explained Common Variance of the general factor = 0.52
##
## Total, General and Subset omega for each subset
## g F1* F2* F3*
## Omega total for total scores and subscales 0.76 NA 0.74 0.53
## Omega general for total scores and subscales 0.52 NA 0.51 0.15
## Omega group for total scores and subscales 0.22 NA 0.24 0.38

### Prosocial

prosocial <- esee %>%
 dplyr::select(sdq_q1, sdq_q4, sdq_q9, sdq_q17, sdq_q20)

summary(omega(prosocial, plot = F))

## Omega
## omega(m = prosocial, plot = F)
## Alpha: 0.68
## G.6: 0.64
## Omega Hierarchical: 0.65
## Omega H asymptotic: 0.92
## Omega Total 0.71
##
## With eigenvalues of:
## g F1* F2* F3*
## 1.504 0.000 0.213 0.092
## The degrees of freedom for the model is -2 and the fit was 0
## The number of observations was 505 with Chi Square = 0 with prob < NA
##
## The root mean square of the residuals is 0
## The df corrected root mean square of the residuals is NA
## Explained Common Variance of the general factor = 0.83
##
## Total, General and Subset omega for each subset
## g F1* F2* F3*
## Omega total for total scores and subscales 0.71 NA 0.43 0.65
## Omega general for total scores and subscales 0.65 NA 0.28 0.65
## Omega group for total scores and subscales 0.04 NA 0.15 0.01

## Model output

summary(factor3model, standardized=TRUE, fit.measures=TRUE)

## lavaan 0.6.16 ended normally after 142 iterations
##
## Estimator ML
## Optimization method NLMINB
## Number of model parameters 75
##
## Number of observations 505
## Number of missing patterns 30
##
## Model Test User Model:
## Standard Scaled
## Test Statistic 945.036 791.828
## Degrees of freedom 249 249
## P-value (Chi-square) 0.000 0.000
## Scaling correction factor 1.193
## Yuan-Bentler correction (Mplus variant)
##
## Model Test Baseline Model:
##
## Test statistic 2113.435 1705.546
## Degrees of freedom 276 276
## P-value 0.000 0.000
## Scaling correction factor 1.239
##
## User Model versus Baseline Model:
##
## Comparative Fit Index (CFI) 0.621 0.620
## Tucker-Lewis Index (TLI) 0.580 0.579
##
## Robust Comparative Fit Index (CFI) 0.644
## Robust Tucker-Lewis Index (TLI) 0.605
##
## Loglikelihood and Information Criteria:
##
## Loglikelihood user model (H0) -7919.409 -7919.409
## Scaling correction factor 2.740
## for the MLR correction
## Loglikelihood unrestricted model (H1) NA NA
## Scaling correction factor 1.551
## for the MLR correction
##
## Akaike (AIC) 15988.819 15988.819
## Bayesian (BIC) 16305.661 16305.661
## Sample-size adjusted Bayesian (SABIC) 16067.603 16067.603
##
## Root Mean Square Error of Approximation:
##
## RMSEA 0.074 0.066
## 90 Percent confidence interval - lower 0.069 0.061
## 90 Percent confidence interval - upper 0.079 0.070
## P-value H_0: RMSEA <= 0.050 0.000 0.000
## P-value H_0: RMSEA >= 0.080 0.034 0.000
##
## Robust RMSEA 0.071
## 90 Percent confidence interval - lower 0.065
## 90 Percent confidence interval - upper 0.077
## P-value H_0: Robust RMSEA <= 0.050 0.000
## P-value H_0: Robust RMSEA >= 0.080 0.006
##
## Standardized Root Mean Square Residual:
##
## SRMR 0.090 0.090
##
## Parameter Estimates:
##
## Standard errors Sandwich
## Information bread Observed
## Observed information based on Hessian
##
## Latent Variables:
## Estimate Std.Err z-value P(>|z|) Std.lv Std.all
## internalising =~
## sdq_q23 1.000 0.170 0.292
## sdq_q19 0.361 0.168 2.146 0.032 0.061 0.309
## sdq_q14 0.297 0.351 0.847 0.397 0.050 0.101
## sdq_q11 -0.021 0.514 -0.040 0.968 -0.003 -0.005
## sdq_q6 1.490 0.421 3.536 0.000 0.253 0.430
## sdq_q24 1.113 0.383 2.905 0.004 0.189 0.443
## sdq_q16 2.207 0.730 3.023 0.003 0.375 0.571
## sdq_q13 0.378 0.196 1.931 0.054 0.064 0.304
## sdq_q8 0.267 0.170 1.572 0.116 0.045 0.249
## sdq_q3 0.229 0.163 1.403 0.161 0.039 0.188
## externalising =~
## sdq_q18 1.000 0.108 0.250
## sdq_q12 0.626 0.176 3.556 0.000 0.067 0.194
## sdq_q7 2.439 0.790 3.088 0.002 0.262 0.466
## sdq_q5 3.125 0.772 4.048 0.000 0.336 0.509
## sdq_q25 2.991 1.068 2.800 0.005 0.322 0.494
## sdq_q21 2.267 0.885 2.562 0.010 0.244 0.387
## sdq_q15 3.766 1.060 3.552 0.000 0.405 0.621
## sdq_q10 3.394 0.894 3.797 0.000 0.365 0.561
## sdq_q2 4.159 1.126 3.693 0.000 0.448 0.620
## prosocial =~
## sdq_q1 1.000 0.341 0.579
## sdq_q4 0.656 0.112 5.883 0.000 0.224 0.382
## sdq_q9 1.326 0.139 9.538 0.000 0.452 0.674
## sdq_q17 0.996 0.119 8.354 0.000 0.340 0.562
## sdq_q20 1.149 0.146 7.865 0.000 0.392 0.539
##
## Covariances:
## Estimate Std.Err z-value P(>|z|) Std.lv Std.all
## internalising ~~
## externalising 0.008 0.003 2.467 0.014 0.463 0.463
## prosocial -0.006 0.009 -0.672 0.502 -0.103 -0.103
## externalising ~~
## prosocial -0.018 0.005 -3.657 0.000 -0.494 -0.494
##
## Intercepts:
## Estimate Std.Err z-value P(>|z|) Std.lv Std.all
## .sdq_q23 0.404 0.026 15.520 0.000 0.404 0.696
## .sdq_q19 0.032 0.009 3.624 0.000 0.032 0.164
## .sdq_q14 0.333 0.022 14.969 0.000 0.333 0.667
## .sdq_q11 0.811 0.033 24.834 0.000 0.811 1.114
## .sdq_q6 0.393 0.026 15.002 0.000 0.393 0.668
## .sdq_q24 0.172 0.019 8.999 0.000 0.172 0.402
## .sdq_q16 0.579 0.029 19.832 0.000 0.579 0.883
## .sdq_q13 0.034 0.009 3.589 0.000 0.034 0.161
## .sdq_q8 0.026 0.008 3.189 0.001 0.026 0.143
## .sdq_q3 0.036 0.009 3.878 0.000 0.036 0.176
## .sdq_q18 0.183 0.019 9.473 0.000 0.183 0.426
## .sdq_q12 0.103 0.015 6.679 0.000 0.103 0.298
## .sdq_q7 0.735 0.025 29.290 0.000 0.735 1.303
## .sdq_q5 0.742 0.029 25.192 0.000 0.742 1.123
## .sdq_q25 1.162 0.029 39.598 0.000 1.162 1.782
## .sdq_q21 1.352 0.028 47.486 0.000 1.352 2.147
## .sdq_q15 0.778 0.029 26.742 0.000 0.778 1.192
## .sdq_q10 0.475 0.029 16.408 0.000 0.475 0.730
## .sdq_q2 0.826 0.032 25.730 0.000 0.826 1.145
## .sdq_q1 1.208 0.026 46.007 0.000 1.208 2.048
## .sdq_q4 1.252 0.026 47.956 0.000 1.252 2.137
## .sdq_q9 1.068 0.030 35.669 0.000 1.068 1.592
## .sdq_q17 1.471 0.027 54.340 0.000 1.471 2.433
## .sdq_q20 0.891 0.033 27.137 0.000 0.891 1.224
## internalising 0.000 0.000 0.000
## externalising 0.000 0.000 0.000
## prosocial 0.000 0.000 0.000
##
## Variances:
## Estimate Std.Err z-value P(>|z|) Std.lv Std.all
## .sdq_q23 0.309 0.023 13.250 0.000 0.309 0.915
## .sdq_q19 0.035 0.011 3.323 0.001 0.035 0.904
## .sdq_q14 0.247 0.016 15.492 0.000 0.247 0.990
## .sdq_q11 0.531 0.023 23.226 0.000 0.531 1.000
## .sdq_q6 0.282 0.026 10.939 0.000 0.282 0.815
## .sdq_q24 0.146 0.021 6.835 0.000 0.146 0.804
## .sdq_q16 0.290 0.049 5.874 0.000 0.290 0.674
## .sdq_q13 0.040 0.012 3.361 0.001 0.040 0.908
## .sdq_q8 0.031 0.011 2.757 0.006 0.031 0.938
## .sdq_q3 0.041 0.012 3.560 0.000 0.041 0.964
## .sdq_q18 0.174 0.019 8.979 0.000 0.174 0.938
## .sdq_q12 0.116 0.020 5.885 0.000 0.116 0.962
## .sdq_q7 0.249 0.018 13.654 0.000 0.249 0.783
## .sdq_q5 0.324 0.023 14.366 0.000 0.324 0.741
## .sdq_q25 0.322 0.028 11.447 0.000 0.322 0.756
## .sdq_q21 0.337 0.026 12.836 0.000 0.337 0.850
## .sdq_q15 0.262 0.023 11.249 0.000 0.262 0.615
## .sdq_q10 0.290 0.028 10.219 0.000 0.290 0.685
## .sdq_q2 0.320 0.028 11.562 0.000 0.320 0.615
## .sdq_q1 0.231 0.019 12.118 0.000 0.231 0.665
## .sdq_q4 0.293 0.019 15.677 0.000 0.293 0.854
## .sdq_q9 0.245 0.024 10.213 0.000 0.245 0.545
## .sdq_q17 0.250 0.023 10.958 0.000 0.250 0.684
## .sdq_q20 0.376 0.028 13.308 0.000 0.376 0.710
## internalising 0.029 0.015 1.942 0.052 1.000 1.000
## externalising 0.012 0.006 1.814 0.070 1.000 1.000
## prosocial 0.116 0.020 5.778 0.000 1.000 1.000

### Robust fit output

fit2 <- cfa(factor3model, data = esee)
fit.robust <- c("chisq.robust", "cfi.robust", "rmsea.robust", "aic")

fitMeasures(factor3model, fit.robust)

## cfi.robust rmsea.robust aic
## 0.644 0.071 15988.819

## Modification indices

modindices(factor3model, sort = TRUE, maximum.number = 5)

## lhs op rhs mi epc sepc.lv sepc.all sepc.nox
## 114 prosocial =~ sdq_q11 75.496 -0.994 -0.339 -0.466 -0.466
## 113 prosocial =~ sdq_q14 69.589 -0.651 -0.222 -0.444 -0.444
## 126 prosocial =~ sdq_q21 56.821 -0.886 -0.302 -0.480 -0.480
## 175 sdq_q14 ~~ sdq_q11 55.855 0.122 0.122 0.336 0.336
## 125 prosocial =~ sdq_q25 50.470 -0.839 -0.286 -0.439 -0.439
